# PUBLIC HEALTH CURRICULUM: DEMENTIA CAREGIVING AS A PUBLIC HEALTH CHALLENGE AND OPPORTUNITY

**DOI:** 10.1093/geroni/igad104.1081

**Published:** 2023-12-21

**Authors:** Eva Jackson, Tara Redd, Elma Johnson, Dionne Bailey, Maya Koffski, Shelby Roberts, Joseph Gaugler

**Affiliations:** Alzheimer’s Association, Chicago, Illinois, United States; Rollins School of Public Health, Emory University, Atlanta, Georgia, United States; University of Minnesota, Minneapolis, Minnesota, United States; University of Minnesota, Minneapolis, Minnesota, United States; University of Minnesota, Minneapolis, Minnesota, United States; Alzheimer’s Association, Chicago, Illinois, United States; University of Minnesota, Minneapolis, Minnesota, United States

## Abstract

A new, free, one-hour, interactive, online module, Dementia Caregiving as a Public Health Challenge and Opportunity, is designed for public health students, educators and professionals. This course approaches caregiving for people with Alzheimer’s disease and other dementias from a population-based perspective , while remembering that these are the individual stories and lives of millions of people today. Dementia caregiving is a public health priority because of its prevalence; its profound influence on individuals, families, communities, and healthcare systems; and its disproportionate effects on subgroups of individuals. A number of public health actions can help to address the population effects of dementia caregiving. There remains a gap in public health curricula that features dementia and dementia caregiving, and this module represents an educational innovation to introduce key concepts in dementia caregiving for students and others. This module defines dementia caregiving, specifies why dementia caregiving is a public health issue, and reviews evidence-based programs for assisting dementia caregivers. Public health examples and case studies are used to support and assess learner understanding. This module was developed for the Alzheimer’s Association by the Emory Centers for Public Health Training and Technical Assistance with support from the Building our Largest Dementia Infrastructure (BOLD) Public Health Center of Excellence on Dementia Caregiving, and represents a novel strategy to elevate dementia caregiving as a matter of public health concern.

